# Identification and Characterization of *PSEUDO-RESPONSE REGULATOR* (*PRR*) *1a* and *1b* Genes by CRISPR/Cas9-Targeted Mutagenesis in Chinese Cabbage (*Brassica rapa* L.)

**DOI:** 10.3390/ijms23136963

**Published:** 2022-06-23

**Authors:** Nan-Sun Kim, Jihyeon Yu, Sangsu Bae, Hyang Suk Kim, Soyoung Park, Kijong Lee, Soo In Lee, Jin A. Kim

**Affiliations:** 1 Department of Agricultural Biotechnology, National Institute of Agricultural Science, Rural Development Administration, Jeonju 54874, Korea; nskims@korea.kr (N.-S.K.); rgtoo1@naver.com (H.S.K.); psy0203@korea.kr (S.P.); leekjong@korea.kr (K.L.); silee@korea.kr (S.I.L.); 2 Division of Life Sciences, Korea Polar Research Institute, Incheon 21990, Korea; muner00@kopri.re.kr; 3 Department of Biomedical Sciences, Seoul National University College of Medicine, Seoul 03080, Korea; sbae7@snu.ac.kr

**Keywords:** CRISPR/Cas9, circadian clock gene, *PSEUDO-RESPONSE REGULATOR 1*, *CIRCADIAN CLOCK-ASSOCIATED 1*, *LATE ELONGATED HYPOCOTYL*, *Brassica rapa* L. ssp. *pekinensis*

## Abstract

The CRISPR/Cas9 site-directed gene-editing system offers great advantages for identifying gene function and crop improvement. The circadian clock measures and conveys day length information to control rhythmic hypocotyl growth in photoperiodic conditions, to achieve optimal fitness, but operates through largely unknown mechanisms. Here, we generated core circadian clock evening components, *Brassica rapa PSEUDO-RESPONSE REGULATOR* (*BrPRR*) *1a*, *1b*, and *1ab* (both *1a* and *1b* double knockout) mutants, using CRISPR/Cas9 genome editing in Chinese cabbage, where 9–16 genetic edited lines of each mutant were obtained. The targeted deep sequencing showed that each mutant had 2–4 different mutation types at the target sites in the *BrPRR1a* and *BrPRR1b* genes. To identify the functions of *BrPRR1a* and *1b* genes, hypocotyl length, and mRNA and protein levels of core circadian clock morning components, *BrCCA1* (*CIRCADIAN CLOCK-ASSOCIATED 1*) and *BrLHY* (*LATE ELONGATED HYPOCOTYL*) *a* and *b* were examined under light/dark cycles and continuous light conditions. The *BrPRR1a* and *1ab* double mutants showed longer hypocotyls, lower core circadian clock morning component mRNA and protein levels, and a shorter circadian rhythm than wildtype (WT). On the other hand, the *BrPRR1b* mutant was not significantly different from WT. These results suggested that two paralogous genes may not be associated with the same regulatory function in Chinese cabbage. Taken together, our results demonstrated that CRISPR/Cas9 is an efficient tool for achieving targeted genome modifications and elucidating the biological functions of circadian clock genes in *B. rapa*, for both breeding and improvement.

## 1. Introduction

Plants have an internal timekeeper known as the circadian clock. The endogenous circadian clock is entrained by the Earth’s 24 h rotation on its axis and plays crucial roles in synchronizing the performance of organisms with daily cycles of light and temperature, enabling plants to regulate activities at the correct time of day [1]. Circadian rhythms are biological cycles that have a period of about 24 h and persist in the absence of external cues. Under natural conditions, circadian rhythms define the daily phase of biological processes, and organize the daily timing of the transcriptome to coordinate cellular processes at appropriate times of day. Circadian regulation increases the performance and fitness of higher plants, including crops [2].

Plant model studies based on molecular genetics using *Arabidopsis thaliana* mutants have revealed that the circadian clock is controlled by the core elements in at least three interlocking loops. *TIMING OF CAB EXPRESSION 1*/*PSEUDO-RESPONSE REGULATOR 1* (*TOC1*/*PRR1*) and two partially redundant *Myb* transcription factors, *CIRCADIAN CLOCK-ASSOCIATED 1* (*CCA1*) and *LATE ELONGATED HYPOCOTYL* (*LHY*), comprise a central oscillation loop [3]. The morning-expressed *Myb* transcription factors, *CCA1* and *LHY*, are often co-expressed and directly bind the evening element in the promoters of *TOC1* to act as transcriptional repressors [4]. TOC1 serves as an evening-expressed protein that is degraded by the photoreceptor F-box protein, *ZEITLUPE* (*ZTL*), before dawn and indirectly induces *CCA1* and *LHY* expression in the morning [5,6]. *TOC1* was the first locus shown to be critical for central components of the circadian clock and mutant phenotype [7]. *TOC1* is one of five members of the *PRR* family, and these proteins are expressed in *Arabidopsis* from morning to night in the following temporal order: *PRR9*, *PRR7*, *PRR5*, *PRR3*, and *TOC1* [8]. Like the other four PRR proteins found in *Arabidopsis*, TOC1 is located in the nucleus and has a pseudo-receiver (PR) domain in the N-terminus and CONSTANS, CONSTANS-LIKE, and *TOC1* (CCT) domains in the C-terminus. The PR domain allows homo- and hetero-oligomerization between the PRRs, and interactions of the PRRs with other proteins [9,10,11]. Through its CCT domain, *TOC1* is able to directly bind DNA, and the PR domain is responsible for transcriptional repression [5].

Although there have been studies of the molecular networks comprising the *Arabidopsis* circadian oscillator, little is known about the precise biochemical and molecular functions of TOC1 within the clock. However, there is evidence that very precise regulation of the *TOC1* gene and protein rhythmic expression are essential for proper clock functions. Different mechanisms contribute to this regulation, including changes in chromatin structure [12], transcriptional regulation [13], and protein degradation by the proteasome pathway [11], which together accurately control the 24 h rhythmic oscillation of *TOC1* expression and function [14]. In *Arabidopsis*, *TOC1* mutant and RNAi plants show a short-period phenotype for clock-controlled gene expression [14,15]. *TOC1* also links environmental information and clock outputs and plays an essential role in constant darkness and in the integration of red-light signals to the clock [11]. The *TOC1* mutants also reduce the responsiveness of hypocotyl elongation and *CCA1* gene expression to red light, suggesting that TOC1 may be involved in phytochrome (phy) signaling to targets other than the clock [11].

The CRISPR/Cas9 genome-editing system has emerged as one of the most advanced systems for engineering crop genomes [16,17]. This technology has developed rapidly, and has been applied to major crops including rice, wheat, and maize, as well as other crops that are important for food security, such as potato and cassava [17,18,19]. In addition, the CRISPR/Cas9 system has proven valuable for rapeseed breeding and improvement [20,21,22,23,24,25]. A few studies have presented results of targeted genome editing mediated by the CRISPR/Cas9 system in *Brassica*, which is an important cruciferous leafy vegetable crop [26,27]. In one of these studies involving the phytoene desaturase gene, *BoPDS*, the S-receptor kinase gene, *BoSRK*, and the male-sterility-associated gene, *BoMS1*, as target genes, multisite and multiple gene mutations were achieved using the endogenous tRNA-processing system to induce highly efficient inheritable mutagenesis in cabbage (*Brassica oleracea* L.) [26].

Chinese cabbage is one of the most important cruciferous leafy vegetables and agronomic crops. It has more complex flower structures than *Arabidopsis*, possibly due to functional diversification of genes in the highly duplicated *Brassica* genome. In previous studies, we showed that the *Brassica rapa* L. *TOC1*/*BrPRR1* gene was present in two copies, *BrPRR1a* and *BrPRR1b*, via Southern blotting and phylogenetic analyses [28,29]. However, the mechanisms of the circadian clock and its components in the *B. rapa* genome are largely unknown. The increase in the temperature of the cultivated area due to global warming affects the productivity of crops, and the Chinese cabbage exposed to high temperature during the cultivation period does not develop heading leaves and shows various nutritional deficiency symptoms. The *TOC1* gene is related to high-temperature morphogenesis and drought response in *Arabidopsis* studies [14,30]. To apply the results of previous studies to overcome environmental stress by regulating the expression of the biological clock gene [31], a study on the function of *TOC1* orthologous genes, *PRR1s*, intrinsic to Chinese cabbage will be helpful. Here, we report application of the CRISPR/Cas9 system to specifically induce mutagenesis of *PRR1a* and *1b* genes, to identify and characterize circadian rhythms in *B. rapa*.

## 2. Results

### 2.1. Selection of Target Genes and Generation of BrPRR1a, 1b, and 1ab Double-Knockout Mutants Using CRISPR/Cas9

To analyze the functions of *BrPRR1a* and *BrPRR1b* genes related to the plant circadian clock in *B. rapa*, the gene sequences were analyzed by NCBI blast. The two genes had a high degree of similarity at both the nucleotide (80.65% identity) and amino acid (84.44% identity) levels (Figure S1) [28]. To apply CRISPR/Cas9 technology to *B. rapa*, target sites for *BrPRR1a*, *BrPRR1b*, and the *BrPRR1a* and *BrPRR1b* double mutation (*BrPRR1ab*) were selected from the exon region of each gene, and a total of 15 single-guide RNAs (sgRNAs) were selected using the CRISPR RGEN tool (https://www.rgenome.net/ accessed on 15 January 2021) (Table 1; Figure S2). 

**Figure 1:**
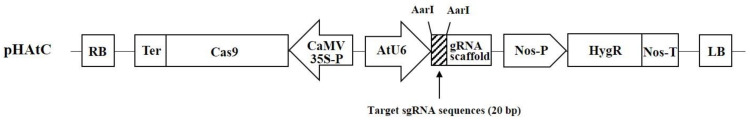
The structure of the T-DNA region of a Cas9/single-guide RNA (sgRNA) plant expression vector, pHAtC. The hygromycin (Hyg) marker gene was driven by the Nos promoter, whereas the sgRNA was driven by the *Arabidopsis* U6 promoter and Cas9 was driven by the CaMV-35S promoter. LB, left border; RB, right border.

Each sgRNA was cloned into the Cas9-sgRNA-expressed plant binary vector, pHAtC, and transformed into *Agrobacterium tumefaciens* GV3101. For transformation, each plant binary vector was transformed into hypocotyl segments of the Chinese cabbage inbred line DH03 (Figure 1).

For each plant binary vector, >16 transgenic plants were regenerated onto selection medium containing hygromycin, and then analyzed in terms of mutation rate to select loss-of-function lines of *BrPRR1a*, *BrPRR1b*, and *BrPRR1ab* mutant T_0_ plants. To identify the sgRNA with high mutation efficiency, NGS analysis was performed using one transgenic T_0_ shoot of each sgRNA in tissue culture vessels. We selected two sgRNA of each gene, BrPRR1a-sgRNA 3 or 4, BrPRR1b-sgRNA 3 or 4, and BrPRR1ab-sgRNA 2 or 3, which showed higher mutation rates than other sgRNA transgenic shoots (Table 2). 

After several transformations using selected sgRNA, 9–16 genetic edited lines of all 6 mutants were obtained. The frequency of insertions and deletions (indels) at each target region ranged from 39% to 100% (Table 3; Table S1). These mutation ratios were similar to those reported in *Arabidopsis* T_1_ mutants [32] and barley T_0_ mutants [33]. 

**Figure 2:**
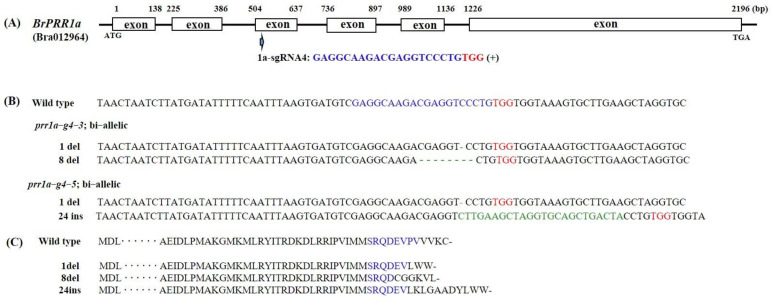
Genome editing of the Chinese cabbage *BrPRR1a* gene. Design of sgRNA sites for *BrPRR1a* exons. The PAM motif (NGG) is shown in red (**A**). Next-generation sequencing (NGS) alignment of the sgRNA target region in different mutant lines. Deletions and insertions are indicated by dashes and green letters, respectively (**B**). Amino acid sequences of wildtype and generated mutations are shown (**C**). The sgRNA and PAM site are represented in blue and red. Stop codon is shown with black dash.

**Figure 3:**
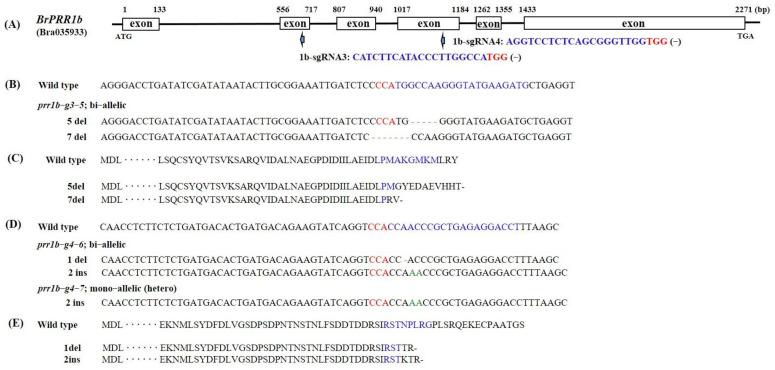
Genome editing of the Chinese cabbage *BrPRR1b* gene. Design of sgRNA sites for *BrPRR1b* exons. The PAM motif (NGG) is shown in red (**A**). NGS alignment of the sgRNA target region in different mutant lines. Deletions and insertions are indicated by dashes and green letters, respectively (**B**,**D**). Amino acid sequences of wildtype and generated mutations are shown (**C**,**E**). The sgRNA and PAM sites are represented in blue and red. Stop codon is shown with black dash.

**Figure 4:**
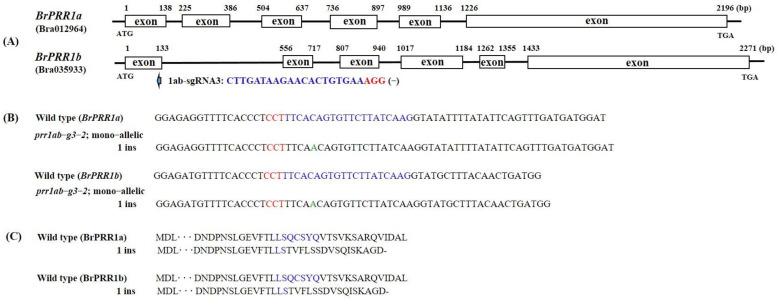
Genome editing of the Chinese cabbage *BrPRR1a* and *1b* genes. Design of sgRNA sites for *BrPRR1a* and *1b* exons. The PAM motif (NGG) is shown in red (**A**). NGS alignment of the sgRNA target region in different mutant lines. Deletions and insertions are indicated by dashes and green letters, respectively (**B**). Amino acid sequences of wildtype and generated mutations are shown (**C**). The sgRNA and PAM sites are represented in blue and red. Stop codon is shown with black dash.

Sequences of each mutated region revealed various types of mutations, including insertions (1–2, 24 bp) and deletions (1, 5, 7–8 bp) of different nucleotides (Figure 2, Figure 3 and Figure 4). These mutants could be divided into homoallelic, heteroallelic, and biallelic or multiallelic mutants. The most common mutations were 1 bp indels (Figure 2B, Figure 3B,D and Figure 4B). The mutations found can lead to formations of premature stop codon at different sites, so truncated protein can be produced (Figure 2C, Figure 3C,E and Figure 4C).

The T_0_ mutant plants were self-pollinated to generate T_1_ progeny seeds. T_1_ progeny seeds were successfully obtained from T_0_ mutant plants with four sgRNAs, but not BrPRR1a-sgRNA3 or BrPRR1ab-sgRNA2. T_1_ mutant plants of BrPRR1a-sgRNA4 (*prr1a-g4*), BrPRR1b-sgRNA3 (*prr1b-g3*) and 4 (*prr1b-g4*), and BrPRR1ab-sgRNA3 (*prr1ab-g3*) were sampled randomly with three replicates and genotyped individually at each target site. The T_1_ progeny plants showed homoallelic, biallelic, and triallelic mutation patterns (Figures S3–S5).

### 2.2. Morphological Effects of BrPRR1a, 1b, and 1ab Mutants in Chinese Cabbage Seedlings

**Figure 5:**
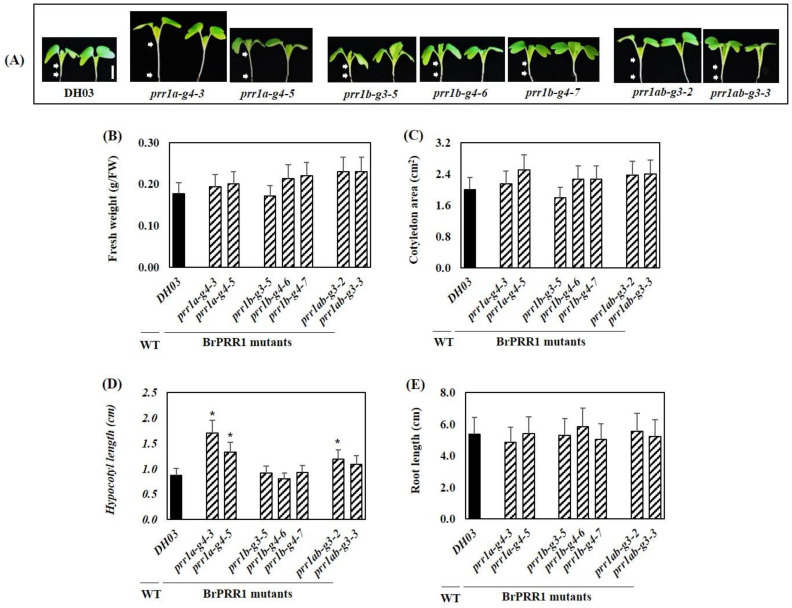
Phenotypes of *B. rapa* seedlings in DH03 and *BrPRR1a*, *1b*, and *1ab* mutants grown under a 16 h/8 h light/dark cycle. Two representative images of each mutant of 8-day-old Chinese cabbage seedlings are shown (**A**). Bar = 1 cm. White arrows indicate the hypocotyl area. Fresh weight (**B**), cotyledon area (**C**), hypocotyl length (**D**), and root length (**E**) of 8-day-old Chinese cabbage sprouts. Values are the means of three biological replicates, and error bars indicate standard deviation (SD). * *p* < 0.05 compared with wildtype control (DH03).

To examine functional conservation of the clock components, several morphological traits of T_1_ mutant lines were compared to the control *B. rapa* inbred line, DH03 (wildtype, WT). Twenty seedlings of each mutant were tested for the phenotype study (Figure S6). Fresh weight and cotyledon area were increased in target gene-mutated lines (except *prr1b-g3-5*) compared to WT. Remarkably, hypocotyls were longer in *prr1a-g4* and *prr1ab-g3* compared to WT, but not in *prr1b-g3* and *prr1b-g4*. The root length was not significantly different between mutated lines and WT (Figure 5). Therefore, the *prr1a-g4* and *prr1ab-g3* lines showed increased fresh weight, cotyledon expansion, and hypocotyl elongation compared to WT. The silencing efficiency of independent lines harboring the CRISPR/Cas9 construct was strongly correlated with the hypocotyl elongation phenotype (Figure 5).

### 2.3. Expression of Circadian Clock Genes in BrPRR1a, 1b, and 1ab Mutants in Chinese Cabbage Seedlings

**Figure 6:**
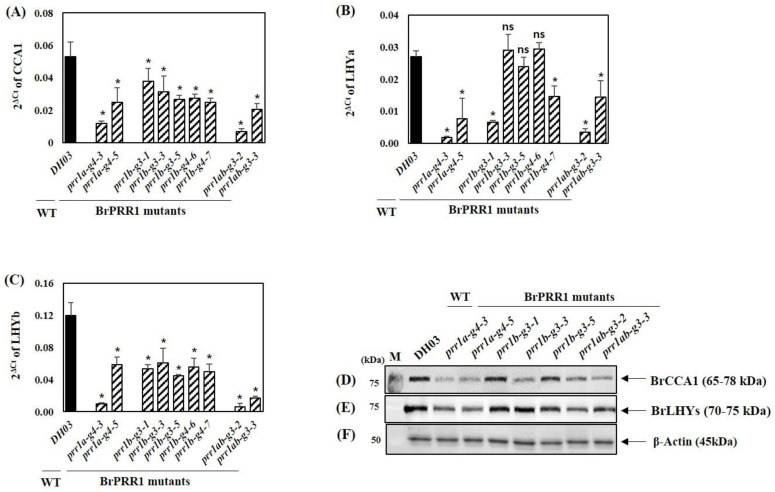
Expression of circadian clock gene in *BrPRR1s* mutants determined by qRT-PCR and Western blotting analysis. To determine the mRNA and protein expression of *BrCCA1* (**A**) and *BrLHYa* (**B**) and *b* (**C**) in DH03 and *BrPRR1a*, *1b*, and *1ab* mutants of Chinese cabbage seedlings, samples were collected at ZT3 for mRNA and protein analysis with liquid nitrogen and stored at −80 °C prior to use. Western blots of 80 μg of total protein extract from WT or BrPRR1 mutants were conducted using anti-CCA1 (**D**) or anti-LHY (**E**), or anti ß-actin polyclonal antibody (**F**) as a primary antibody and anti-rabbit HRP conjugated antibody as a secondary antibody. ß-actin was used for loading controls. Values are the means of three biological replicates, and error bars indicate standard deviation (SD). * *p* < 0.05 and ns = not significant compared with the wildtype control (DH03).

In a previous study, we identified candidate paralogs of circadian clock-related genes and assessed their expression levels in Chinese cabbage by RNA-sequencing (RNA-Seq) [34]. Based on RNA-Seq data, the *B. rapa* genome contains three *MYB* transcription factor genes, a single copy of the *BrCCA1* gene, and two copies of the *BrLHY* gene (*BrLHYa* and *BrLHYb*), which play key regulatory roles in the endogenous clock [3,4]. RNA-Seq data revealed that *BrCCA1* and *BrLHYa* and *b* transcripts under light/dark (LD) conditions peaked at Zeitgeber time (ZT) 0 [34]. To examine the *BrCCA1* and *BrLHYa* and *b* gene expression levels in *BrPRR1* mutant lines, total RNA and proteins were analyzed in 8-day-old Chinese cabbage seedlings grown under a 16 h light/8 h dark cycle at ZT3 (Figure 6). Quantification of mRNA expression was performed by quantitative real-time PCR (qRT-PCR) using gene-specific primers (Table S4). In most of the *BrPRR1* mutants, the *BrCCA1* and *BrLHYa* and *b* mRNA expression levels were lower than in the WT (Figure 6A–C).

Remarkably, the mRNA and protein expression levels of *BrCCA1* and *BrLHYa* and *b* were reduced in *prr1a-g4* and *prr1ab-g3* lines compared to the WT. The *prr1b-g3* and *prr1b-g4* lines showed varying *BrCCA1* and *BrLHYa* and *b* mRNA and protein expression levels (Figure 6A–F).

We found markedly decreased *BrCCA1* and *BrLHYa* and *b* mRNA and protein expression in BrPRR1a-related mutants compared to the WT. Additionally, we analyzed the protein expression of BrPRR1s of WT or BrPRR1 mutants at ZT12, which is the peak time point of BrPRR1s [34] using the anti-TOC1 polyclonal antibody. BrPRR1s protein expression in all BrPRR1 mutants decreased compared to the WT, especially in *prr1a-g4-3* and *prr1ab-g3-2* (Figure S7). Therefore, we chose *prr1a-g4-3*, *prr1b-g3-5*, and *prr1ab-g3-2* for further experiments.

### 2.4. BrPRR1 Mutant Changed the Regulation of BrCCA1 and BrLHYa and b Expression in Chinese Cabbage

**Figure 7:**
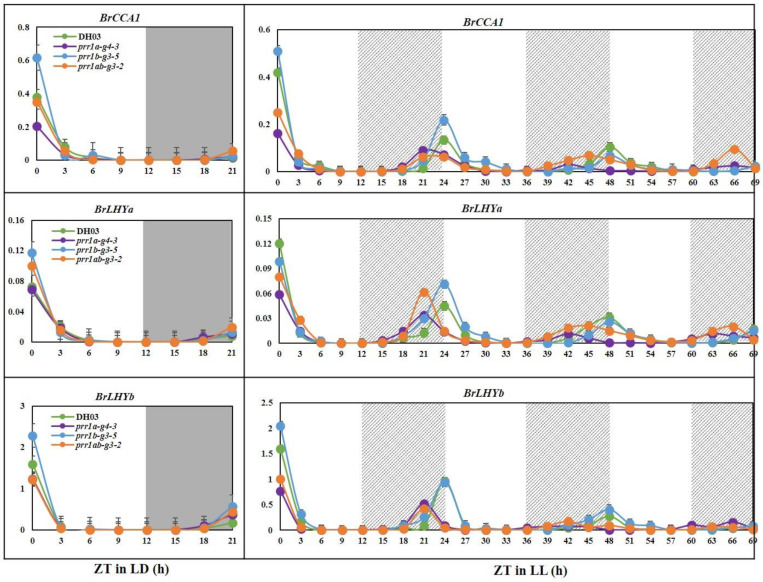
Expression of *BrCCA1* and *BrLHYa* and *b* under different light regimens. The leaves of 2-week-old plants grown in soil were harvested at the indicated times for total RNA extraction and qRT-PCR analysis. Data are the mean ± SD of three biological replicates. Diurnal rhythm of *BrCCA1* and *BrLH*Ya and *b* mRNA abundance under LD (16 h/8 h) conditions. Circadian rhythm of the mRNA expression of *BrCCA1* and *BrLHYa* and *b* in plants grown under LD conditions and transferred to constant light (LL). Time is presented as Zeitgeber time (ZT), in which the dark to light transition is defined as ZT0. Gray regions indicate nighttime. Shaded regions indicate subjective night, defined by the entraining light/dark cycle.

We examined the expression of core circadian clock elements, *BrCCA1* and *BrLHYa* and *b*, in *BrPRR1* mutants. To examine the circadian rhythms of the selected clock genes in *B. rapa*, we analyzed candidate gene transcript accumulation in plants grown under LD and continuous light (LL) conditions. Three transgenic plants and WT (DH03) grown under LD for 1 day and under LL for 3 days were harvested every 3 h. Quantification of mRNA expression was performed by qRT-PCR using gene-specific primers (Table S4). *BrCCA1* and *BrLHYa* and *b* transcripts were largely unchanged under LD and LL, although their levels of expression were lower in the *prr1a-g4-3* and *prr1ab-g3-2* mutants than WT, except for *prr1b-g3-5* (Figure 7). Interestingly, *BrCCA1* and *BrLHYa* and *b* transcripts showed clear circadian rhythm with a peak at ZT21 under LL in the *prr1a-g4-3* and *prr1ab-g3-2* mutants, while WT and *prr1b-g3-5* peaked at ZT24. These results indicated that the period of circadian-related gene expression in the *prr1a-g4-3* and *prr1ab-g3-2* mutants was shortened by an average of 3 h compared to WT and *prr1b-g3-5* (Figure 7).

## 3. Discussion

### 3.1. Conservation of Genes through Evolution and Obtaining New Functions

Genes can be conserved with paralogous copies through whole-genome duplications and segmental duplications. These duplicated genes can either lose (pseudogenization) or gain functions (neofunctionalization) [35]. Most orthologous genes retain the same function among species and their paralogous copies show redundant functions; however, some genes show clear evidence of sub-functionalization, such as the MADs-box gene family related to flowering development in plants [36]. In *A. thaliana*, the variation of glucosinolates related to resistance to generalist insects is due in part to the tandemly duplicated methylthioalkylmalate synthase (*MAM*) genes, and the *MAM2* locus is a stronger deterrent against generalist herbivores than the *MAM1* locus [37]. The acquisition or loss of function of genes involved in plant development and reproduction (for example) during evolution is not only of academic interest, it is also directly relevant to the productivity of crops and can have effects on the human diet and economy.

### 3.2. PRR Genes in Plants

Among the five *Arabidopsis* clock-associated *PRR* genes (*PRR1*, *PRR3*, *PRR5*, *PRR7*, and *PRR9*), the *PSEUDO-RESPONSE REGULATOR* (*PRR*) plays a key role as the main oscillator in the multiple feedback loop, along with the *CCA1*/*LHY* families [13,15,38]. In *B. rapa*, the PRR family has 11 members after genome triplication, and microsynteny analysis suggested that they have a significantly higher rate of retention than their neighboring genes [39]. Some PRR members produced as a result of rearrangements and partial deletions showed very low expression levels or different expression patterns [40]. It is still not known whether these different paralogous copies are functional or nonfunctional and, if they are active, whether they are redundant or neofunctional. Previous studies indicated that the mRNA sequence of *BrPRR1b* is more divergent from *PRR1* than *BrPRR1a*. *BrPRR1a* and *BrPRR1b* are 87.3% and 83.1% identical, respectively, to *PRR1*, and the exon–intron structure of *BrPRR1b* is different from that of the other two genes [34]. In addition, the expression of the two *PRR1* orthologs peaked at different time points and their expression levels were different. These results suggest that there may be a change in the circadian rhythm regulatory mechanism of *B. rapa* containing *PRR*. Mutant studies are useful to determine the functions of genes. We constructed *BrPRR1a* and *BrPRR1b* mutants and *BrPRR1a* and *BrPRR1b* double mutants using site-directed gene editing. *Arabidopsis* toc1 mutants show shortened circadian rhythms (~21 h) under LD conditions and long hypocotyl phenotypes [7,15]. While the hypocotyl length of *BrPRR1b* mutants was similar to WT, *BrPRR1a* mutants and *BrPRR1ab* double mutants showed long hypocotyls compared to WT (Figure 5). These results suggest that *BrPRR1a* may be the main orthologous gene of *Arabidopsis TOC1*, while *BrPRR1b* may be nonfunctional or neofunctional. As the mRNAs of both paralogous genes are expressed with similar rhythms and at similar levels, studying the function of the *PRR* gene will be helpful to determine the specific mechanism of circadian rhythm regulation in Chinese cabbage, and should provide information allowing improvement of agricultural traits using the circadian clock genes. The functions of the two paralogous genes do not completely overlap, and the mutants developed in this study will be useful to study the functions of *BrPRR1s* and mechanisms of circadian rhythm regulation that have evolved specifically in *B. rapa*.

### 3.3. Possibility of Using PRR Genes in Crop Breeding

Transcriptomics and metabolomics studies revealed that many genes related to the photosystem, photosynthesis, and various key secondary metabolite pathways are controlled by clock genes [41]. The circadian clock is important for increasing plant biomass, photosynthetic capacity, and survival [42]. The circadian clock regulates responses to abiotic stress [43,44,45,46,47], and circadian rhythms and their regulatory mechanisms are key factors in the ecological properties of plants. Recently, through the development of gene-editing technology, attempts have been made to modulate the clock genes of cultivated plants, rather than their metabolism or adaptability to environmental stresses [31,48,49,50,51].

TOC1 has attracted attention due to its association with drought stress response mechanisms, via reciprocal regulation between *ABAR* and *TOC1* [14]. It was also demonstrated that the interaction between *TOC1* and *PIF4* mediates circadian gating of thermoresponsive growth, indicating that the *TOC1* gene is involved in the mechanism of high-temperature stress resistance [30]. In recent studies, TOC1 was shown to contribute to the diurnal regulation of metabolism by binding directly to the promoter of the TCA-related gene *FUMARASE 2*, to repress its expression at night [52]. Improvements in source capacity, alterations in plant architecture, and increased resistance to abiotic and biotic stresses could improve crop yields. The relations of *TOC1* with the abiotic stress response and primary metabolism suggest a role for the *TOC1* gene in successful crop breeding. *BrPRR1s* gene-edited Chinese cabbage mutants will be useful in research aiming to improve many agricultural traits involving the PRR genes.

## 4. Materials and Methods

### 4.1. Plant Materials and Growth Conditions

The inbred line DH03 (*B. rapa* L. ssp. *pekinensis*) was used in this study. Seedlings were grown on soil at 23 °C in a growth chamber with a controlled environment, under a 16 h/8 h LD cycle for 8 days and with cool-white fluorescent illumination (100 mol m^−2^ s^−1^, FLR40D/A fluorescent tube; Osram, Seoul, Korea). For circadian period determination, 2-week-old plants were entrained under a 12 h/12 h LD cycle for 1 week before transfer to LL conditions. The plants were watered every 3 days, and temperature was maintained at 23 °C until the emergence of 3–5 true leaves. Aerial parts of 3-week-old plants were harvested at ZT0, 3, 6, 9, 12, 15, 18, and 21, with three biological replicates [34].

### 4.2. CRISPR/Cas9 Target Site Selection and Vector Construction

For targeting *PRR1* genes in *B. rapa* L., guide RNA target sequences were selected from the coding sequence of *BrPRR1a* (Bra012964, GenBank accession No. GU219472) and *BrPRR1b* (Bra035933, GenBank accession no. GU219477) using Cas-designer (http://www.rgenome.net/cas-designer/ accessed on 15 January 2021). Only gRNA sequences without an off-target containing one or two mismatches were selected during target selection to minimize the off-target effect. Off-target sites of each guide RNA in the *B. rapa* genome were predicted using Cas-OFFinder (http://www.rgenome.net/cas-offinder/ accessed on 15 January 2021). Predicted off-targets with 1–2 bp mismatch did not exist, and few off-targets with mismatch 3 bp were found (Table S2). Although, sequences and position of predicted off-target sites with 3 bp mismatch were presented (Table S3). Common guide RNAs of *BrPRR1* that could target both *PRR1a* and *PRR1b* genes were selected in the conserved sequences of the two genes (Table 1). A pair of oligonucleotides containing 20 bp target sequences of each guide RNA were annealed for target cloning. Annealed oligos were cloned into the *Aar*I site of the plant binary vector for CRISPR-editing, i.e., pHAtC (kindly provided by Jin-Soo Kim; Addgene plasmid #78098) [53], which contains a codon-optimized Cas9 driven by a CaMV-35S promoter and sgRNA scaffold directed by the *Arabidopsis* U6 promoter. The Ti-plasmid vectors for sgRNA expression, *BrPRR1a*, *BrPRR1b*, and *BrPRR1ab*/pHAtC, were mobilized into *Agrobacterium tumefaciens* strain GV3101.

### 4.3. Chinese Cabbage Transformation and Acclimation to the Greenhouse

Chinese cabbage was transformed as described previously [31]. Hypocotyls were cut into 0.5 cm segments and placed on pre-culture medium {Murashige and Skoog (MS) basal medium (Duchefa, Harrlem, The Netherlands) + 1 mg/L naphthylacetic acid (NAA, Duchefa) + 3 mg/L benzyladenine (BA, Duchefa) +2 mg/L AgNO_3_ (Sigma, St Louis, MO, USA) + 3% sucrose (Duchefa) + 0.9% phyto agar (Duchefa)}. The pre-cultured hypocotyls were inoculated with *Agrobacterium* suspension in MS liquid medium for 15–20 min. The inoculated hypocotyls were then placed on co-cultivation medium (MS basal medium + 1 mg/L NAA + 3 mg/L BA + 3% sucrose + 0.9% phyto agar) and incubated for 3 days in the dark at 23 °C. To remove *Agrobacterium*, the explants were washed three to five times in liquid co-cultivation medium supplemented with 100 mg/L of carbenicillin (Duchefa) and 250 mg/L of cefotaxime (Duchefa) and transferred to selective medium (MS basal medium + 1 mg/L NAA + 3 mg/L BA + 2 mg/L AgNO_3_ + 10 mg/L hygromycin (Duchefa) + 3% sucrose + 100 mg/L carbenicillin + 250 mg/L cefotaxime + 0.9% phyto agar). Calli that formed on the hypocotyls were sub-cultured on fresh selective medium, which was replaced every 2–3 weeks until the shoots regenerated. The regenerated shoots were transferred to MS medium without plant growth hormones to induce root formation. To confirm the transgene, independent transformed lines were analyzed by PCR. The transformed plants were grown in a greenhouse after acclimation.

### 4.4. Targeted Deep Sequencing and Mutation Analysis

Genomic DNA was extracted from the leaves of transgenic *B. rapa* using the DNA Quick Plant Kit (GeneAll, Seoul, Korea). PCR was performed to amplify the genomic region containing the CRISPR/Cas9 target sites using adaptor primers (Table S4) with Phusion High-Fidelity DNA polymerase (New England Biolabs, Ipswich, MA, USA). The resulting PCR amplicons were subjected to paired-end sequencing with the Illumina MiniSeq system (Illumina, San Diego, CA, USA). After sequencing, paired-end reads were joined by Fastq-join in ea-utils (https://code.google.com/archive/p/ea-utils/ accessed on 15 January 2021) using the default parameters. Paired-end reads were then analyzed by comparing WT and mutant sequences using the Cas-Analyzer (http://www.rgenome.net/cas-analyzer/ accessed on 15 January 2021) [54]. Insertions and deletions around the Cas9 cleavage site (3 bp upstream of the protospacer-adjacent motif sequence) were considered as mutations induced by Cas9.

### 4.5. Quantitative Real-Time PCR Expression Analysis in B. rapa

Quantitative real-time PCR (qRT-PCR) analysis was performed with 100 ng of cDNA in a 20 μL reaction volume using AccuPower^®^ 2× GreenStar™ qPCR Master Mix (Bioneer, Oakland, CA, USA). The gene-specific primers are listed in Table S4. qRT-PCR was performed with an initial step at 95 °C for 10 min followed by 40 cycles of 95 °C for 20 s, 58 °C for 20 s, and 72 °C for 25 s. Fluorescence was recorded after the last step of every cycle. Three replicates were performed per sample. Amplification, data processing, and detection were performed using the CFX96™ Real-Time PCR Detection System (Bio-Rad, Hercules, CA, USA). Quantification cycle (cq) values were examined using the 2^−ΔCT^ method to determine changes in gene expression.

### 4.6. Western Blotting Analysis

Proteins were extracted from 0.5 g of ground power from frozen tissue with 0.5 mL of extraction buffer {50 mM Tris-HCl (Sigma), pH 7.5, 150 mM NaCl (Sigma), 1 mM EDTA (Sigma), 0.5% NP-40 (Sigma), 1 mM DTT (Sigma), 2 mM Na_3_VO_4_ (Sigma), 2 mM NaF (Sigma), 50 µM MG132 (Sigma)} containing a tablet of Complete Protease Inhibitor Cocktail (Roche, Basel, Switzerland), and centrifuged at 15,000× *g* for 20 min to remove debris. For Western blotting analysis, 80 µg of total protein extract was separated by 8% (*w*/*v*) SDS-PAGE and electroblotted onto polyvinylidene difluoride (PVDF) membranes (Millipore, Temecula, CA, USA). The membranes were then incubated in blocking solution (5% (*w*/*v*) non-fat dried milk in TBST buffer {20 mM Tris-Cl, pH 7.5, 500 mM NaCl, and 0.05% Tween 20 (Sigma)}, followed by rabbit polyclonal antibody to CCA1 (Agrisera, Vannas, Sweden) or LHY (Agrisera), or TOC1 (Abiocode, Agoura hills, CA, USA), and then horseradish peroxidase (HRP, Sigma)-conjugated anti-rabbit IgG as a secondary antibody. ß-actin was used for loading controls. The protein concentration was determined using a DC Protein Assay Kit (Bio-Rad), with bovine serum albumin as the standard.

### 4.7. Statistical Analysis

For phenotype and gene expression studies, twelve and three seedlings of T_1_ mutants were used, respectively. Data are expressed as mean ± standard deviation (SD) of three biological replicates. Statistical analysis was performed with Student’s *t* test. In all analyses, *p* < 0.05 was taken to indicate statistical significance.

## Figures and Tables

**Table 1:** List of selected sgRNAs targeting the *BrPRR1a* and *BrPRR1b* genes and potential off-target sites in the *B. rapa* genome, determined using CRISPR RGEN tools (http://www.regenome.net/cas-designer/ accessed on 15 January 2021).

Gene	sgRNA	sgRNA Target	Direction	GC Contents (%)	Out-of Frame Score	Mismatches		
						0 bp	1 bp	2 bp
*BrPRR1a*	1a-sgRNA1	TGATCCCAATAGCTTGGGAGAGG	+	50	70.2	1	0	0
	1a-sgRNA2	AAAACCTCTCCCAAGCTATTGGG	−	40	73.4	1	0	0
	1a-sgRNA3	GCCTGACATCGATATAATACTGG	+	40	56.4	1	0	0
	1a-sgRNA4	GAGGCAAGACGAGGTCCCTGTGG	+	65	52.3	1	0	0
	1a-sgRNA5	TCAAGCACTTTACCACCACAGGG	+	45	60.1	1	0	0
*BrPRR1b*	1b-sgRNA1	GGAGGGTGAAAACATCTCCCAGG	−	55	56.5	1	0	0
	1b-sgRNA2	TGCGGAAATTGATCTCCCCATGG	+	50	72.1	1	0	0
	1b-sgRNA3	CATCTTCATACCCTTGGCCATGG	−	50	57.4	1	0	0
	1b-sgRNA4	AGGTCCTCTCAGCGGGTTGGTGG	−	65	80.8	1	0	0
	1b-sgRNA5	CGGCTTAAAGGTCCTCTCAGCGG	−	55	72.6	1	0	0
*BrPRR1a* and *BrPRR1b* (*1ab*)	1ab-sgRNA1	TTATTGACAGAAGCAGAGTTAGG	+	35	60.5	2	0	0
	1ab-sgRNA2	ATAAGAACACTGTGAAAGGAGGG	−	35	85.4	2	0	0
	1ab-sgRNA3	CTTGATAAGAACACTGTGAAAGG	−	35	78.3	2	0	0
	1ab-sgRNA4	TCACACGTGACAAAGATCTTCGG	+	40	61.7	2	0	0
	1ab-sgRNA5	CCAACGAGCTTCTCAACTTGTGG	+	50	76.5	2	0	0

**Table 2:** The indel frequencies of each target region in T_0_ mutants of Chinese cabbage by NGS-based targeted deep sequencing.

Gene	sgRNA	Target Region	Sample	Total	Insertion	Deletion	Mutated Count	Mutated Ratio
*BrPRR1a*	1a-sgRNA1	TGATCCCAATAGCTTGGGAGAGG	*prr1a-g1*	30,969	1505	397	1902	6.14%
	1a-sgRNA2	AAAACCTCTCCCAAGCTATTGGG	*prr1a-g2*	30,205	7706	421	8127	26.91%
	1a-sgRNA3	GCCTGACATCGATATAATACTGG	*prr1a-g3*	8436	0	8428	8428	99.91%
	1a-sgRNA4	GAGGCAAGACGAGGTCCCTGTGG	*prr1a-g4*	11,652	0	11,652	11,652	100.00%
	1a-sgRNA5	TCAAGCACTTTACCACCACAGGG	*prr1a-g5*	37,980	463	537	1000	2.63%
*BrPRR1b*	1b-sgRNA1	GGAGGGTGAAAACATCTCCCAGG	*prr1b-g1*	25,547	80	46	126	0.49%
	1b-sgRNA2	TGCGGAAATTGATCTCCCCATGG	*prr1b-g2*	41,371	2424	1315	3739	9.04%
	1b-sgRNA3	CATCTTCATACCCTTGGCCATGG	*prr1b-g3*	9903	3	9898	9901	99.98%
	1b-sgRNA4	AGGTCCTCTCAGCGGGTTGGTGG	*prr1b-g4*	6633	9	3432	3441	51.88%
	1b-sgRNA5	CGGCTTAAAGGTCCTCTCAGCGG	*prr1b-g5*	41,905	431	231	662	1.58%
*BrPRR1ab*		*BrPRR1a* region						
	1ab-sgRNA1	TTATTGACAGAAGCAGAGTTAGG	*prr1ab-g1*	12,299	2978	210	3188	25.92%
	1ab-sgRNA2	ATAAGAACACTGTGAAAGGAGGG	*prr1ab-g2*	16,666	7618	8945	16,563	99.38%
	1ab-sgRNA3	CTTGATAAGAACACTGTGAAAGG	*prr1ab-g3*	16,353	3858	3480	7338	44.87%
	1ab-sgRNA4	TCACACGTGACAAAGATCTTCGG	*prr1ab-g4*	35,791	0	0	0	0.00%
	1ab-sgRNA5	CCAACGAGCTTCTCAACTTGTGG	*prr1ab-g5*	25,968	40	75	115	0.44%
		*BrPRR1b* region						
	1ab-gRNA1	TTATTGACAGAAGCAGAGTTAGG	*prr1ab-g1*	13,192	3299	9887	13,186	99.95%
	1ab-sgRNA2	ATAAGAACACTGTGAAAGGAGGG	*prr1ab-g2*	18,464	8607	9818	18,425	99.79%
	1ab-sgRNA3	CTTGATAAGAACACTGTGAAAGG	*prr1ab-g3*	15,966	8329	6763	15,092	94.53%
	1ab-sgRNA4	TCACACGTGACAAAGATCTTCGG	*prr1ab-g4*	37,652	0	3	3	0.01%
	1ab-sgRNA5	CCAACGAGCTTCTCAACTTGTGG	*prr1ab-g5*	31,533	69	136	205	0.65%

**Table 3:** Mutation frequencies of *BrPRR1a* and *1b* genes in T_0_ mutants of Chinese cabbage determined using the CRISPR/Cas9 system.

Sample	No. of Genome Mutated Plants	Indel Frequency (%)	Genotypes (No.)				
			Homo Allelic	Hetero Allelic	Bi Allelic	Multiple Allelic	Mosaic Allelic
*prr1a-g3*	13	99.8–100			1		12
*prr1a-g4*	16	99.9–100			14	2	
*prr1b-g3*	11	98.3–100	2		5	2	2
*prr1b-g4*	13	44.9–100		2	8	1	2
*prr1ab-g2*	9	51–99.9	1	2	5	1	
*prr1ab-g3*	13	39–99.9		5		2	6

## Data Availability

This manuscript includes the necessary data either as figures or as Supplementary Materials.

## Supplementary Materials

The following supporting information can be downloaded at: https://www.mdpi.com/article/10.3390/ijms23136963/s1.
