# Supplementary material for: Identification and Characterization of PSEUDO-RESPONSE REGULATOR (PRR) 1a and 1b Genes by CRISPR/Cas9-Targeted Mutagenesis in Chinese Cabbage (Brassica rapa L.)
Source: Int J Mol Sci. 2022 Jun 23;23(13):6963. doi: 10.3390/ijms23136963 (PMC9266808; doi:10.3390/ijms23136963)
Supplement: Supplementary file 1 [file ijms-23-06963-s001.zip › ijms-1728819-supplementary/Revision-supplementary figures.pdf]

Supplementary Materials:

(A)

```
TOC1         1  ATGGATTTGAACGGTGAGTGTAAAGGAGGAG------ATGGGTTTATTGATAGAAGCAGAGTCAGGATTTTGCTTTGTGACAATGATTCCACGAGTTTGGGAGAGGTTTTTACTCTCCTT
BrPRR1b      1  ATGGATTTGAACGGTGAGTGTAAGGGAGGAG------ATGGGTTTATTGACAGAAGCAGAGTTAGGATTTTGCTTTGTGACAATGATTCCAAGAGCCTGGGAGATGTTTTCACCCTCCTT
BrPRR1a      1  ATGGATTTGAACGGAGAGTGTAAGGGAGGAGGAGGAGATGGGTTTATTGACAGAAGCAGAGTTAGGATTTTGCTTTGTGACAATGATCCCAATAGCTTGGGAGAGGTTTTCACCCTCCTT
                **************:********.*******      ************* *********** ************************ ***. **  ******* ***** ** ******

TOC1       121  TCAGAGTGTTCTTATCAAGTGACTGCAGTGAAATCAGCAAGGCAGGTGATTGATGCACTTAATGCAGAGGGACCTGATATCGATATAATACTGGCGGAAATTGATCTCCCAATGGCTAAG
BrPRR1b    121  TCACAGTGTTCTTATCAAGTGACTTCAGTGAAATCAGCAAGGCAGGTGATTGATGCACTAAACGCAGAGGGACCTGATATCGATATAATACTTGCGGAAATTGATCTCCCCATGGCCAAG
BrPRR1a    121  TCACAGTGTTCTTATCAAGTGACGTCAGTCAAATCAGCAAGGCAGGTGATTGATGCACTGAATGCAGAGGGGCCTGACATCGATATAATACTGGCGGAGATTGATCTCCCAATGGCCAAG
                *** *******************  **** ****************************** ** ********.***** ************** *****.***********.***** ***

TOC1       241  GGTATGAAGATGCTGAGGTACATCACACGAGACAAAGATCTTCGCAGAATCCCTGTGATAATGATGTCGAGGCAAGACGAAGTCCCTGTCGTTGTAAAGTGCTTGAAGCTAGGTGCAGCT
BrPRR1b    241  GGTATGAAGATGCTGAGGTACATCACACGTGACAAAGATCTTCGGAGAATCCCTGTCATAATGATGTCGAGGCAAGACGAGGTACCTGTTGTGGTGAAGTGCTTGAAGCTAGGTGCAGCT
BrPRR1a    241  GGTATGAAGATGCTAAGGTACATCACACGTGACAAAGATCTTCGGAGAATCCCTGTCATCATGATGTCGAGGCAAGACGAGGTCCCTGTGGTGGTAAAGTGCTTGAAGCTAGGTGCAGCT
                **************.**************:************** *********** **.*******************.**.***** ** **.************************

TOC1       361  GACTACCTTGTGAAGCCTCTTCGCACCAACGAGCTTCTGAACTTGTGGACACACATGTGGAGAAGAAGACGCATGCTAGGACTTGCTGAGAAGAATATGTTGAGCTATGATTTTGATCTT
BrPRR1b    361  GACTACCTTGTCAAGCCTCTTCGCACCAACGAGCTTCTCAACTTGTGGACATACATGTGGAGAAGAAGACGCATGCTAGGACTTGCTGAGAAGAATATGTTGAGCTATGATTTTGATCTT
BrPRR1a    361  GACTACCTTGTGAAGCCTCTTCGGACCAACGAGCTTCTCAACTTGTGGACACACATGTGGAGAAGAAGACGCATGCTAGGACTTGCTGAGAAGAACATGTTGAGCTATGAGTTCGATCTC
                *********** *********** ************** ************ ******************************************** ************** ** *****

TOC1       481  GTGGGATCTGATCAAAGTGATCCAAACACAAATAGTACCAACCTGTTCTCTGACGACACAGATGATAGAAGTCTTAGGTCCACCAACCCACAGAGAGGAAATTTAAGTCACCAGGAAAAT
BrPRR1b    481  GTGGGATCTGATCCAAGTGATCCAAACACAAACAGTACCAACCTCTTCTCTGATGACACTGATGACAGAAGTATCAGGTCCACCAACCCGCTGAGAGGACCTTTAAGCCGTCAGGAAAAA
BrPRR1a    481  GTGGGATCTGACCCAAGTGATCCAAATACAAACAGCACCAACCTCTTCTCTGATGACACAGATGAGAGAAGTATTAGGTCCACCAATCCGCAGAGAG------GAAGTCATCAGGAAAAG
                *********** *.************ ***** ** ******** ******** *****:***** ******.* *********** **.*:*****      *** *. ********

TOC1       501  GAGTGGTCTGTTGCTACTGCTCCTGTTCATGCTCGTGATGGTGGTCTTGGTGCTGATGGAACAGCCACTTCTTCTCTTGCTGTTACTGCTATAGAGCCTCCATTGGATCATCTTGCTGGG
BrPRR1b    501  GAGTGTCCTGCTGCTACTGGTTCTG--------------------------TTGATGGCACAGCCACTTCAGCTCCT-----------------CCTCCATTAAATCATCTTCCTGGG
BrPRR1a    501  GAGTGGCCTGTTCCTACTGGTTCCGTTTGTGCTGGCGATGGTG------CTGCTGATGGCACAGCCACGTCAACTCCTCCCGTTGCTATCATAGAGCCTCCTTTGAATCATCTTCCTGAA
                *****  *** * ****** * * *                           ******.******** **: *** *                  *****:**..******** ***..

TOC1       621  TCTCACCATGAGCCAATGAAAAGAAATAGTAATCCAGCGCAATTTTCTTCAGCACCGAAGAAAAGTAGATTGAAGATCGGAGAGTCCTCTGCTTTCTTTACATATGTCAAATCTACTGTC
BrPRR1b    621  TCTCATCATGAGCCTAA---------------TCCAGAGAAATTTTCTTCAGTGCCAAAGAAGAGTAGATTGAAGATTGGAGAGTCCTCAGCTTTCTTCACATATGTCAAGTCTACCGCC
BrPRR1a    621  CCTCACCATGAGCCTACCAAAAGAAATACTAATCCAGCGCAATTTTCTTCAGTACCAAAGAAGAGTAGATTGAAGATTGGAGAGTCCTCCGCTTTCTTCACATATGTCAAGTCTACT---
                 **** ********:*                *****.*.************ .**.*****.************** *********** ******** ***********.*****

TOC1       741  CTTAGAACTAACGGTCAGGATCCTCCTCTTGTCGATGGAAATGGCTCACTTCATCTTCATCGGGGTTTGGCGGAGAAGTTTCAAGTGGTGGCTAGTGAAGGGATCAACAACACCAAACAA
BrPRR1b    741  CTGGCTACCAACTGTCAAGATCCTCCCCATGTCAATGGAAACGGCTCACTTCATCTTCACCCAGGTGTG---------------------------------------------------
BrPRR1a    741  -----------------------------GTCAATGGAAATGGCTCAGTTCATCCAGGTATG--------------------------------------------------------
                                              ***.******* ****** ****** : .. .

TOC1       861  GCACGCAGAGCAACACCAAAATCTACTGTCCTTAGAACTAACGGTCAGGATCCTCCTCTTGTCAATGGAAATGGCTCACATCATCTTCATCGGGGTGCGGCGGAAAAGTTTCAAGTGGTG
BrPRR1b    861  ---------------------------------------------------------------------------------------------GTTGCGGAGAAGCTTCAAGTGGTG
BrPRR1a    861  ------------------------------------------------------------------------------------------------GCTGAGAAGCTTCAAGCGGTG
                                                                                                               ** **.*** ****** ****

TOC1       981  GCTAGTGAAGGGATCAACAACACCAAACAAGCACACAGAAGTAGAGGGACCGAGCAATACCATTCTCAAGGAGAGACCTTGCAGAATGGCGCCAGCTATCCACATTCCCTTGAGCGGTCA
BrPRR1b    981  GCCAGTGAGGTGATCAACAAGCCCAAGCAAACACACCGAAGCAGAGAGACTGAG-----------------AAAAACTTACAGAATGGCGGCG-----------------------
BrPRR1a    981  GCAAGTGAGGTGATCAACAACGCCAAACAAACACGAGGAGGTAGAGAGACTGAGAAAAACAAAGCTCAAGGAGAGAACTTAGTGAACGGCACGCTTG--------------AGCGGTCA
                ** *****.* *********  ****.***.***.. **.* ****.*** ***                  .*.*.***. :*** ***.

TOC1      1001  CGCACGCTTCCCACATCAATGGAATCTCATG---GTAGGAACTACCAAGAGG--------------------------------------------------------GCAATATGAATATT
BrPRR1b   1001  -------------CCACGGAAGAACTTCATG---GTAGGAGTTACCAAGAG-------------------------------------------------------------------AGG
BrPRR1a   1001  CGCACGCTTCCAACACCAATGGAACTTCATGGCAGTAGGAGTTACCAAGAAGTTCCAAATTCTATTGAGCGGTCACGCACGCTTCCACCACCAATGGAACTTCACGGTACTAGGAGTTGT
                             *. *..:.***  *****   ******. ********.                                                                  :

TOC1      1121  CCCCAAGTTG---CTATGAACAGAAGTAAAGATTCGTCTCAAGTTGATGGATCGGGTTTCTCTGCACCAAATGCCTATCCTTACTATATGCATGGGGTCATGAACCAAGTTATGATGCAA
BrPRR1b   1121  AATCAGGTTG---CTGTGAACAGAAGTAAGGATTCATCTCAAGTT--------------------------GCCTATCCTTACTATATGCAAGGGGTCATGAACCAAGTTATGATGCAA
BrPRR1a   1121  TACCAAGAAGGATCTATGGATGATGCCCGGGTTGCTGCTGCTAAGGATTCATCTCAGTTCCCTGCGCAAAATGCCTATCCTTACTATATGCATGGTGTCATGAACCAAGTTATGATGCAA
                 . **.*::*   **.**.* ..:.  ...*:* *  ** .:.:                          ********************:** ***********************

TOC1      1141  TCAGCAGCCATGATGCCTCAATATG---GTCATCAAATTCCTCATTGCCAACCAAATCATCCGAATGGAATGACGGGATATCCT---TACTACCACCACCCAAT---GAACACATCTTTG
BrPRR1b   1141  TCAGCAGCCATGATGCCTCAGTATGGTCATCATCAACATCCTCATTACCCACCTAATCATCTCAATGGAATGACAGGAGTTCCTTATTACCACCACCATCCCAT---GAACACACCCTTA
BrPRR1a   1141  TCAGCAGCCATGATGCCTCAGTATGGTCATCATCAACATCCTCACTGCCCGCCTAATCATCTGAATGGCATGACAGGGTTTCCTTATTACCACCACCACCACCAGATGAACACATCGTTA
                ********************.****   .*******.:****** *.**..**:*******  *****.*****.**. :****   *** ******* *...:   ******* * **.

TOC1      1261  CAGCATAGTCAGATGTCTTTACAGAATGGTCAGATG---TCTATGGTTCATCATTCTTGGTCACCGGCAGGA---------AATCCGCCTTCTAATGAGGTGAGGGTAAATAAACTTGAC
BrPRR1b   1261  CAGCACAATCAAATG---TCACAGAATGGTCAGATG---TCTATGGTTCATTAT-------------------------CATCCGTCATCTAACGAGGTGAGAGCGAGTAAACTTGAC
BrPRR1a   1261  CAGAACGGTCATGTGCCTTTACAGAATGGTCAGATGCCTCCTATGGTTCATCATCATCATTCTTGGCCACAGGTGGGAAACCACCCGTCTCCTAACGAGGTGAGAGTGACTAAGCTTGAC
                ***.* ..*** .**   * ****************   *********** **                          .* *** *: **** ********.* .* ***.******

TOC1      1381  AGAAGAGAGGAAGCTCTGCTGAAATTCAGACGTAAAAGGAACCAACGTTGTTTTGATAAGAAGATTAGGTATGTGAATAGGAAACGCCTTGCTGAGAGGAGACCCCGCGTTAAGGGTCAG
BrPRR1b   1381  AGAAGAGAGGAAGCTTTGCTTAAATTTAGACGTAAGAGGAACCAAAGGTGTTTTGATAAGAAGATTAGGTATGTGAATAGGAAGAAGCTCGCTGAGAGGAGGCCGCGTGTTAAGGGTCAG
BrPRR1a   1381  AGAAGAGAGGAAGCGTTGCTTAAATTTAGACGTAAGAGGAACCAAAGGTGTTTTGATAAGAAGATTAGGTATGTGAATAGGAAGAAGCTTGCTGAGAGGAGGCCACGTGTTAAGGGTCAG
                **************  **** ***** ********.*********.* ***********************************... ** ***********.** ** ************

TOC1      1501  TTTGTTAGGAAGATGAACGGCGTGAATGTTGATTTAAATGGACAGCCTGACTCT------GCTGACTATGATGACGAGGAAGAGGAGGAAGAAGAAGAAGAAGAGGAGAACCGGGATTCA
BrPRR1b   1501  TTTGTTAGGAAAATGAACGGGGTGAATGTTGACTTAAATGGGCAGCCT--------------GACTATGATGACGAGGAAGAGGAGGAGGATGAAGAGGAAGAGGAGAATAGGGACTCA
BrPRR1a   1501  TTTGTTAGGAAGATGAACGGCGTAAATGTTGACTTGAACGGGCAGCCTGAACCTGACTCTGCTGACTATGATGACGAGGAAGAGGAG---GATGAAGAAGAAGAGGAGAATCGGGACTCG
                ***********.******** **.******** **.** **.******              ************************   **:*****.*********** .**** **.

TOC1      1621  TCTCCTCAGGATGATGCTTTGGGAACTTGA
BrPRR1b   1621  TCTCCTCAGGATGATGCTCAGGGAACTTGA
BrPRR1a   1621  TCTCCTCAGGATGATGCTCTTGGGACTTGA
                ****************** : **.******
```

(B)

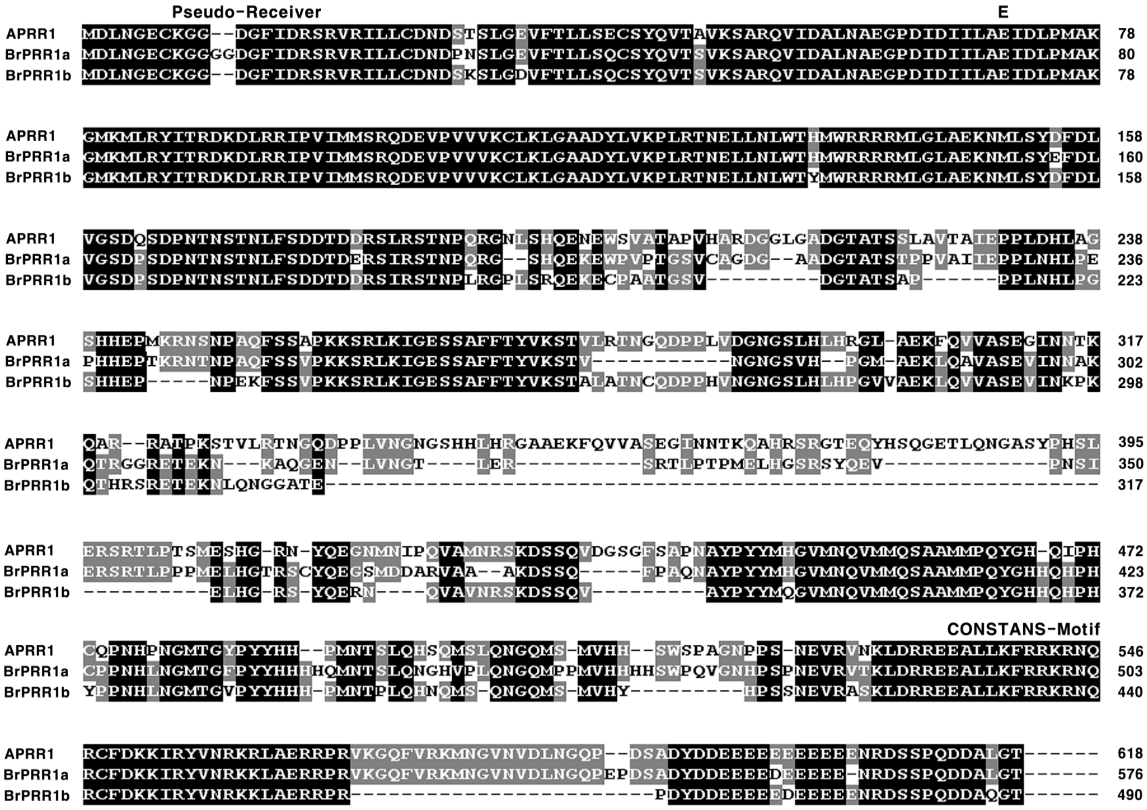

Figure S1. Comparison of *Arabidopsis PRR1* and its *B. rapa* orthologs *BrPRR1a* and *BrPRR1b*. Alignment of mRNA (A) and amino acid sequences [28] (B).

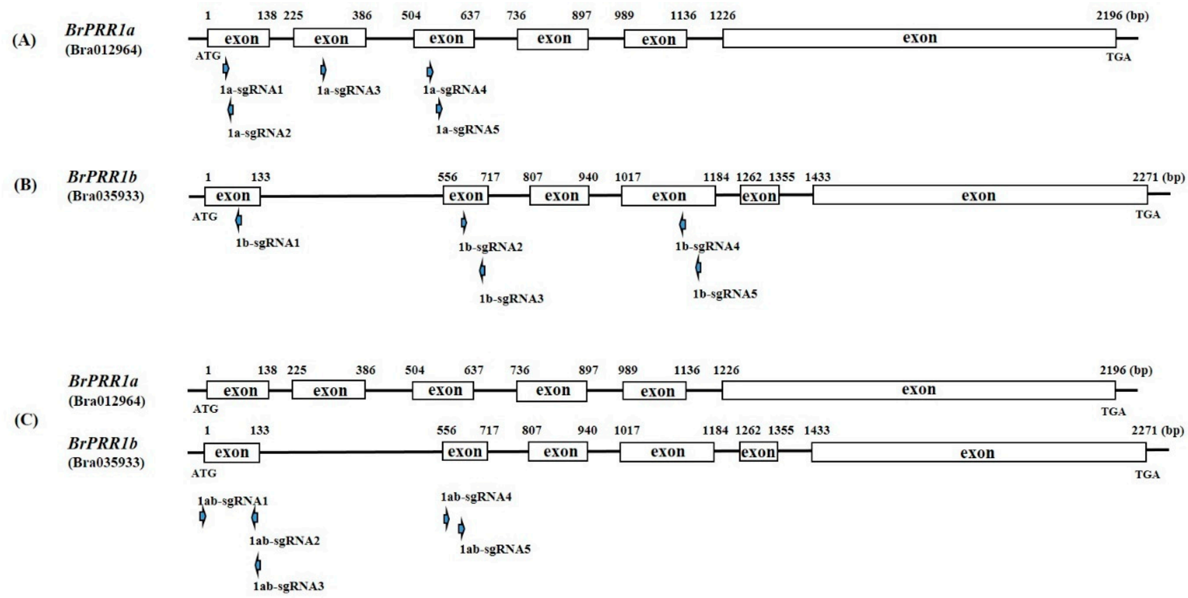

Figure S2. Schematic maps of the sgRNA target sites of *BrPRR1a* and *BrPRR1b* in *B.rapa* genome. Arrows show the positions of sgRNA target site for *BrPRR1a* (A), *BrPRR1b* (B), and *BrPRR1a* and *BrPRR1b* double mutations (C).

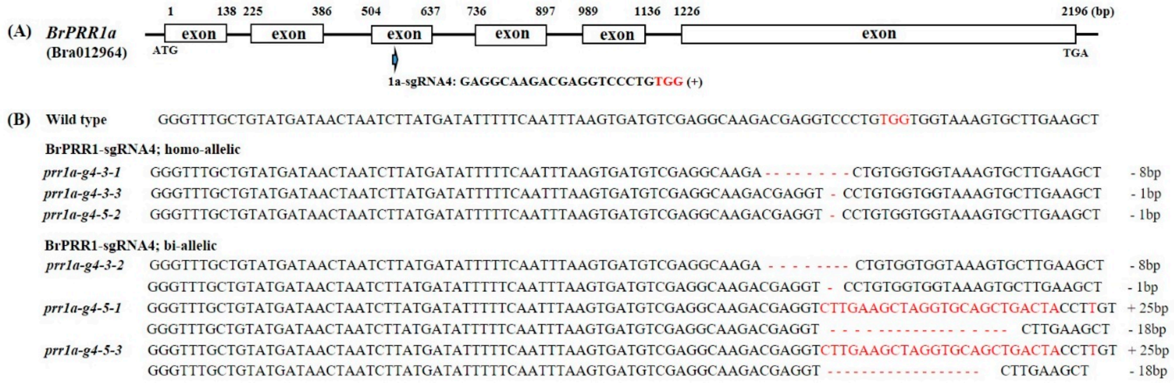

Figure S3. Genome editing of the Chinese cabbage *BrPRR1a* gene. Design of sgRNA site for *BrPRR1a* exons. The PAM motif (NGG) is shown in red (A). Next-generation sequencing (NGS) alignment of the sgRNA target region in different mutant lines. Deletions and insertions are indicated by dashes and red letters, respectively (B).

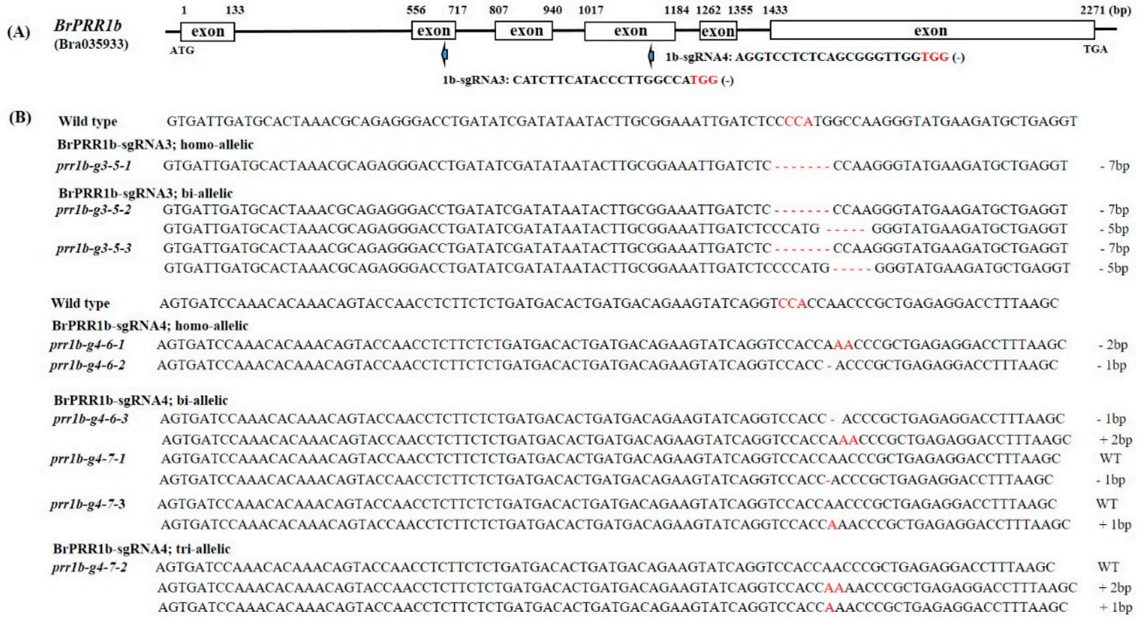

Figure S4. Genome editing of the Chinese cabbage *BrPRR1b* gene. Design of sgRNA sites for *BrPRR1b* exons. The PAM motif (NGG) is shown in red (A). Next-generation sequencing (NGS) alignment of the sgRNA target region in different mutant lines. Deletions and insertions are indicated by dashes and red letters, respectively (B).

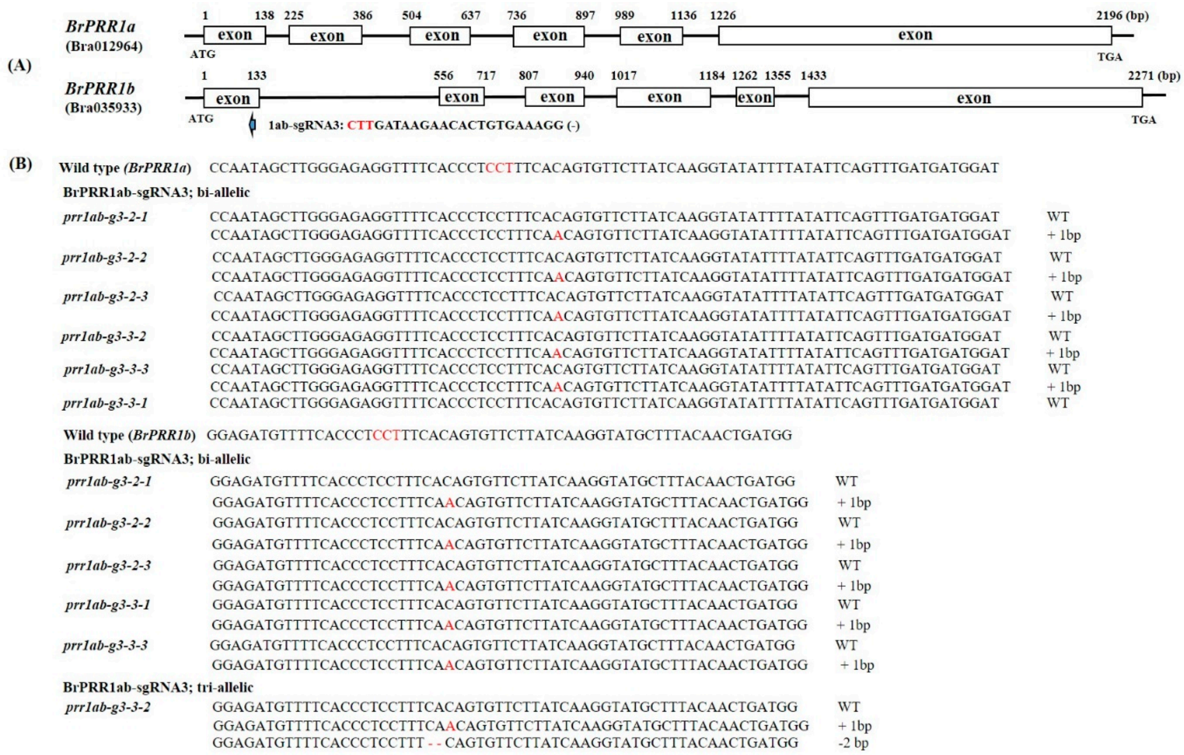

Figure S5. Genome editing of the Chinese cabbage *BrPRR1a* and *1b* genes. Design of sgRNA site for *BrPRR1a* and *1b* exons. The PAM motif (NGG) is shown in red (A). NGS alignment of the sgRNA target region in different mutant lines. Deletions and insertions are indicated by dashes and red letters, respectively (B).

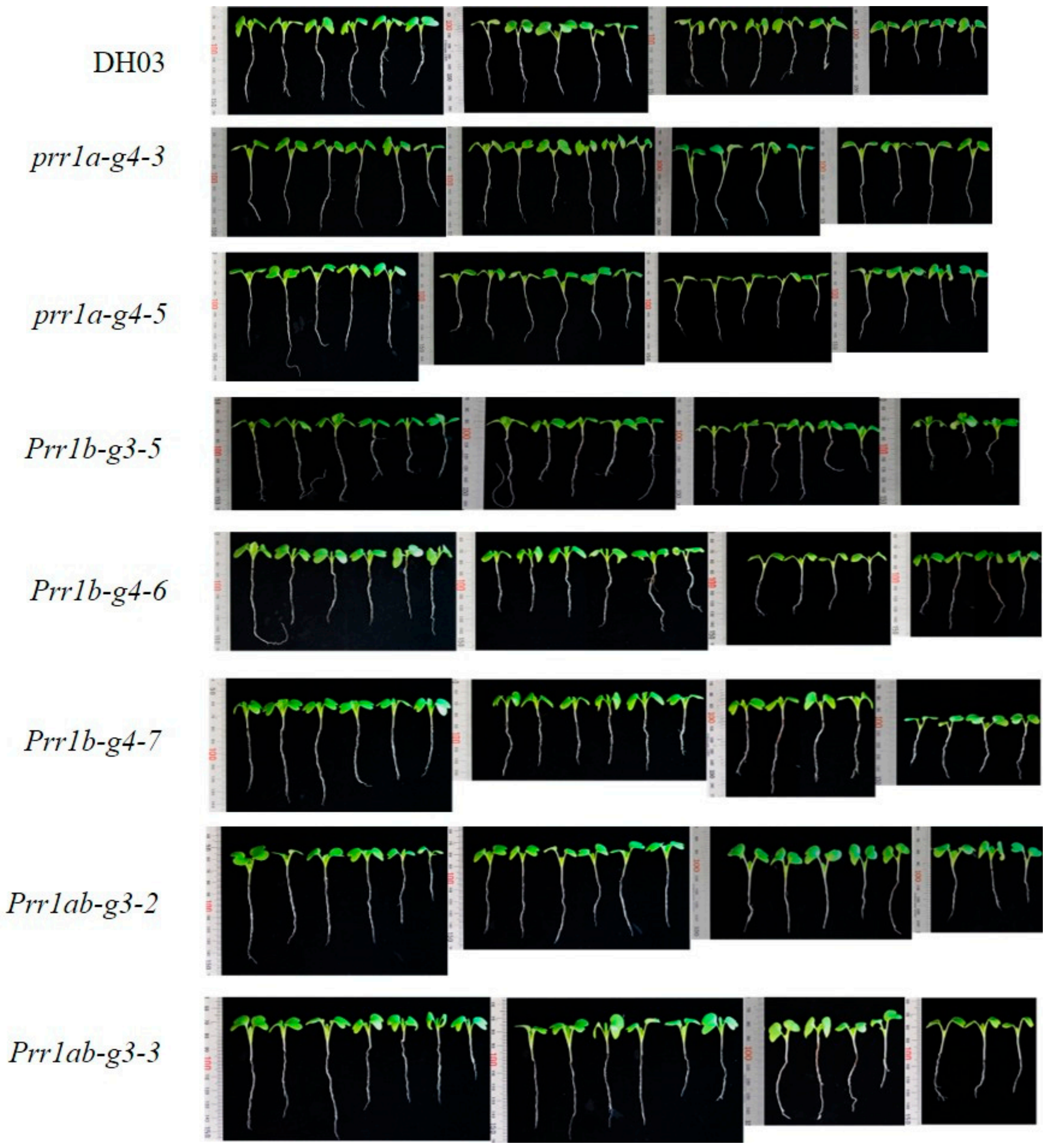

Figure S6. Phenotypes of 20 seedlings of BrPRR1a, BrPRR1b, and BrPRR1ab T_1 mutants in Chinese cabbage.

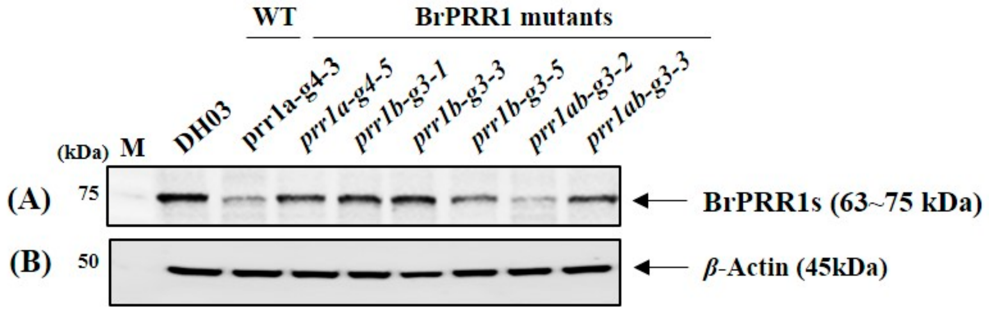

Figure S7. Protein expression of BrPRR1s in BrPRR1 mutants determined by Western blotting analysis. To determine the protein expression in BrPRR1a, BrPRR1b, and BrPRR1ab mutants, samples were collected at ZT12 with liquid nitrogen and at −80°C prior to use. 80μg of total protein extract was separated by 8% SDS-PAGE and electroblotted onto PVDF membranes. Primary and secondary antibody were used rabbit anti-TOC1 polyclonal antibody (Abiocode, Agoura hills, CA, USA) and goat anti-rabbit HRP conjugated antibody (A). *β*-actin was used for loading controls (B).
