# Supplementary material for: Identification and Characterization of PSEUDO-RESPONSE REGULATOR (PRR) 1a and 1b Genes by CRISPR/Cas9-Targeted Mutagenesis in Chinese Cabbage (Brassica rapa L.)
Source: Int J Mol Sci. 2022 Jun 23;23(13):6963. doi: 10.3390/ijms23136963 (PMC9266808; doi:10.3390/ijms23136963)
Supplement: Supplementary file 1 [file ijms-23-06963-s001.zip › ijms-1728819-supplementary/Revision-supplementary table.pdf]

Table S1. Mutation frequency of *BrPRR1a*, *BrPRR1b* and *BrPRR1ab* genes using CRISPR/Cas9 system.

**Target region**	**Sample**	**Total count**	**Insertion**	**Deletion**	**Indel**	**Indel frequency**	**Genotype**	**Mutation analysis**							
	prr1a-g3-1	8436	0	8428	8428	99.91%	bi-allelic	1 del	65.20%	2 del	33.30%				
	prr1a-g3-2	7464	568	6896	7464	100.00%	hetero	1 del	49.30%	mosaic					
	prr1a-g3-3	7035	0	7035	7035	100.00%	mosaic								
	prr1a-g3-4	6794	0	6794	6794	100.00%	mosaic								
	prr1a-g3-5	7661	0	7661	7661	100.00%	mosaic								
	prr1a-g3-6	7594	1043	6551	7594	100.00%	mosaic								
**BrPRR1a-sgRNA3**	*prr1a-g3-7*	6344	0	6344	6344	100.00%	mosaic								
	prr1a-g3-8	6976	0	6976	6976	100.00%	mosaic								
	prr1a-g3-9	5856	0	5856	5856	100.00%	mosaic								
	prr1a-g3-10	7159	0	7159	7159	100.00%	mosaic								
	prr1a-g3-11	7626	0	7626	7626	100.00%	mosaic								
	prr1a-g3-12	7436	539	6897	7436	100.00%	mosaic								
	prr1a-g3-13	7062	558	6504	7062	100.00%	mosaic								
	prr1a-g4-1	8972	2959	6013	8972	100.00%	bi-allelic	1 del	63.50%	24 ins	34.20%				
	prr1a-g4-2	10321	0	10321	10321	100.00%	bi-allelic	8 del	53.00%	1 del	44.80%				
	prr1a-g4-3	11652	0	11652	11652	100.00%	bi-allelic	8 del	52.20%	1del	45.90%				
	prr1a-g4-4	10359	0	10353	10353	99.94%	bi-allelic	8 del	51.90%	1 del	45.90%				
	prr1a-g4-5	8369	2844	5525	8369	100.00%	bi-allelic	1 del	62.60%	24 ins	35.50%				
	prr1a-g4-6	9061	3138	5923	9061	100.00%	bi-allelic	1 del	61.80%	24 ins	35.90%				
	prr1a-g4-7	10651	2	10649	10651	100.00%	bi-allelic	8 del	50.90%	1 del	46.90%				
**BrPRR1a-sgRNA4**	*prr1a-g4-8*	10917	2001	8916	10917	100.00%	tri-allelic	1 del	52.80%	12 del	27.00%	1 ins	18.00%		
	prr1a-g4-9	10564	0	10564	10564	100.00%	bi-allelic	26 del	58.10%	9 del	39.60%				
	prr1a-g4-10	10303	0	10303	10303	100.00%	bi-allelic	26 del	55.70%	9 del	42.00%				
	prr1a-g4-11	9916	0	9916	9916	100.00%	bi-allelic	26 del	57.90%	9 del	39.50%				
	prr1a-g4-12	7381	3952	3424	7376	99.93%	Quad-allelic	1 ins	35.30%	7 del	26.40%	1 del	19.20%	1 ins	17.60%
	prr1a-g4-13	10180	0	10180	10180	100.00%	bi-allelic	26 del	59.10%	9 del	38.60%				
	prr1a-g4-14	9433	2	9431	9433	100.00%	bi-allelic	26 del	67.80%	9 del	30.10%				
	prr1a-g4-15	7528	3589	3935	7524	99.95%	bi-allelic	2 del	51.30%	1 ins	46.70%				
	prr1a-g4-16	6734	3221	3511	6732	99.97%	bi-allelic	2 del	51.40%	1 ins	47.10%				

Continued

Target region	Sample	Total count	Insertion	Deletion	Indel	Indel frequency	Genotype	Mutation pattern					
**BrPRR1b-sgRNA3**	*prr1b-g3-1*	9903	3	9898	9901	99.98%	homo	1 del	99.10%				
	prr1b-g3-2	10586	0	10584	10584	99.98%	homo	1 del	98.10%				
	prr1b-g3-3	10074	0	10072	10072	99.98%	bi-allelic	5 del	49.50%	7 del	48.40%		
	prr1b-g3-4	10069	0	10069	10069	100.00%	bi-allelic	5 del	48.90%	7 del	48.70%		
	prr1b-g3-5	8573	0	8569	8569	99.95%	bi-allelic	7 del	49.70%	5 del	48.80%		
	prr1b-g3-6	9467	0	9467	9467	100.00%	bi-allelic	5 del	49.80%	7 del	48.40%		
	prr1b-g3-7	9447	4071	5368	9439	99.92%	hexa-allelic	13 del	22.40%	5 del	17.80%	1 del	15.60%
								1 ins	15.10%	1 ins	14.30%	1 ins	13.40%
	prr1b-g3-8	7780	3176	4573	7749	99.60%	mosaic						
	prr1b-g3-9	8373	3200	5170	8370	99.96%	hexa-allelic	1 ins	25.20%	13 del	19.50%	3 del	14.30%
								1 del	14.20%	1 del	13.00%	1 ins	12.60%
	prr1b-g3-10	6337	2530	3733	6263	98.83%	mosaic						
	prr1b-g3-11	9467	0	9467	9467	100.00%	bi-allelic	5 del	49.80%	7 del	48.40%		
**BrPRR1b-sgRNA4**	*prr1b-g4-1*	6633	9	3432	3441	51.88%	hetero	1 del	50.80%				
	prr1b-g4-2	6289	6268	2	6270	99.70%	bi-allelic	1 ins	50.70%	1 ins	47.60%		
	prr1b-g4-3	6733	4415	2310	6725	99.88%	tri-allelic	1 ins	43.20%	1 del	33.70%	1 ins	21.45%
	prr1b-g4-4	6504	0	6502	6502	99.97%	bi-allelic	21 del	49.30%	9 del	48.70%		
	prr1b-g4-5	10358	10346	2	10348	99.90%	bi-allelic	1 ins	49.20%	1 ins	49.20%		
	prr1b-g4-6	12878	6238	6636	12874	99.97%	bi-allelic	1 del	50.50%	2 ins	47.80%		
	prr1b-g4-7	11300	5545	4	5549	49.11%	hetero	1 ins	48.50%				
	prr1b-g4-8	8103	0	8093	8093	99.88%	bi-allelic	21 del	54.20%	7 del	44.00%		
	prr1b-g4-9	8283	0	8283	8283	100.00%	bi-allelic	21 del	56.20%	7 del	42.10%		
	prr1b-g4-10	7291	623	6668	7291	100.00%	mosaic						
	prr1b-g4-11	4732	1967	2765	4732	100.00%	mosaic						
	prr1b-g4-12	4341	431	3910	4341	100.00%	mosaic						
	prr1b-g4-13	6504	0	6502	6502	99.97%	bi-allelic	21 del	49.30%	9 del	48.70%		

Continued

Target region	Sample	Total count	Insertion	Deletion	Indel	Indel frequency	Genotype	Mutation pattern					
**BrPRR1ab -sgRNA2**	*prr1ab-g2-1*	5908	3702	2201	5903	99.92%	bi-allelic	1 ins	61.30%	5 del	36.90%		
	prr1ab-g2-2	6429	6417	5	6422	99.89%	homo	1 ins	98.50%				
	prr1ab-g2-3	7552	4424	3124	7548	99.95%	bi-allelic	1 ins	58.10%	5 del	41.00%		
	prr1ab-g2-4	7363	4341	3015	7356	99.90%	bi-allelic	1 ins	58.10%	5 del	40.50%		
	prr1ab-g2-5	7542	4340	3188	7528	99.81%	bi-allelic	1 ins	56.80%	5 del	41.60%		
	prr1ab-g2-6	6818	3864	2949	6813	99.93%	bi-allelic	1 ins	56.10%	5 del	42.80%		
	prr1ab-g2-7	1206	804	303	1107	91.79%	hetero	1 ins	52.30%				
	prr1ab-g2-8	2915	1464	24	1488	51.05%	hetero	1 ins	49.50%				
	prr1ab-g2-9	11319	3397	7908	11305	99.88%	tri-allelic	2 del	36.70%	1 del	32.10%	1 ins	29.60%
**BrPRR1ab -sgRNA3**	*prr1ab-g3-1*	10773	26	6546	6572	61.00%	hetero	14 del	35.70%				
	prr1ab-g3-2	10969	4507	4052	8559	78.03%	hetero	1 ins	40.10%				
	prr1ab-g3-3	11992	6186	0	6186	51.58%	hetero	1 ins	50.40%				
	prr1ab-g3-4	9181	4354	4710	9064	98.73%	mosaic	10 del	37.00%				
	prr1ab-g3-5	8555	3567	2798	6365	74.40%	hetero	1 ins	39.80%				
	prr1ab-g3-6	9095	3723	5363	9086	99.90%	tri-allelic	1 ins	39.90%	14 del	33.40%	6 del	24.60%
	prr1ab-g3-7	9642	4194	5446	9640	99.98%	tri-allelic	1 ins	42.20%	14 del	31.20%	6 del	24.30%
	prr1ab-g3-8	10980	41	4240	4281	39.0%	mixed						
	prr1ab-g3-9	9379	4292	1797	6089	64.92%	hetero	1 ins	42.90%				
	prr1ab-g3-10	1468	31	542	573	39.03%	mixed						
	prr1ab-g3-11	1572	238	875	1113	70.80%	mixed	12 del	32.70%				
	prr1ab-g3-12	1107	546	465	1011	91.33%	mixed	1 ins	49.80%	14 del	24.60%		
	prr1ab-g3-13	716	640	71	711	99.30%	mixed	1 ins	41.30%	1 ins	33.40%		

Table S2. Predicted Off-target sites of each guide RNA in *B. rapa* genome using Cas-OFFinder (http://www.rgenome.net/cas-offinder/) used in this study.

Label	Sequences	Predicted off-target sites				
		1	2	3	4	mismatch
**BrPRR1a-sgRNA3**	GCCTGACATCGATATAATACTGG	0	0	0	0	
**BrPRR1a-sgRNA4**	GAGGCAAGACGAGGTCCCTGTGG	0	0	0	3	
**BrPRR1b-sgRNA3**	CATCTTCATACCCTTGGCCATGG	0	0	1	12	
**BrPRR1b-sgRNA4**	AGGTCCTCTCAGCGGGTTGGTGG	0	0	0	4	
**BrPRR1ab-sgRNA2**	ATAAGAACACTGTGAAAGGAGGG	0	0	2	22	
**BrPRR1ab-sgRNA3**	CTTGATAAGAACACTGTGAAAGG	0	0	2	21	

Table S3. Sequences and position of predicted off-target sites with 3 bp mismatch.

Label	Sequences	Position in genome		Direction
**BrPRR1b-sgRNA3-off1**	CATCTTCATACCaaTGGCCtTGG	A06	2820332	-
**BrPRR1ab-sgRNA2-off1**	ATAAcAACAaTGTGAAAGGgTGG	A09	28541205	+
**BrPRR1ab-sgRNA2-off2**	AaAAGAACAtTGTGAAAGaAAGG	A09	35155954	-
**BrPRR1ab-sgRNA3-off1**	CTTGcTAAGAACACTGTttAAGG	A01	19835080	+
**BrPRR1ab-sgRNA3-off2**	aTTGAgAAGAACACTGTtAAAGG	A08	10887388	-

Red low letters represented mismatch with gRNA and underbar showed seed region of gRNA.

Table S4. List of primers used in this study

Primer name	DNA sequence (5'-3')	Purpose
ß-actin F	TGGCATCACACTTTTCTACAA	RT-qPCR
ß-actin R	CAACGGAATCTCTCAGCTCC	
BrCCA1 F	TCTCTGTCACATGCTCCTCCTT	
BrCCA1 R	CGGCTAAGTTCCCTTGTGG	
BrLHYa F	AGACATTCATGGAATCTTCTG	
BrLHYa R	GCGGTAATCATGCTGGAAAG	
BrLHYb F	CTGTATGCGCTCCTCCTGCT	
BrLHYb R	TTGTCTTTGAGCCAGCGTTG	
1a-sgRNA4 1st F	GTTTGAGCTGCTATCGATTGTT	Targeted deep-sequencing
1a-sgRNA4 1st R	AAGCATTGAGCGCAAACTAAG	
1a-sgRNA4 2nd F	ACACTCTTTCCCTACACGAC GCTCTTCCGATCT CATTTACTTTCTCTACTTAGCAAAG	
1a-sgRNA4 2nd R	GTGACTGGAGTTCAGACGTGT GCTCTTCCGATCT GCATAGAGAGAACTAAAGATAGA	
1b-sgRNA3 1st F	AATGCATATGTAGTGTCTATTGCA	Targeted deep-sequencing
1b-sgRNA3 1st R	TAGCATTCACAGCAACAAAGCT	
1b-sgRNA3 2nd F	ACACTCTTTCCCTACACGAC GCTCTTCCGATCT CTTGAAACGCAGTGACTTCAG	
1b-sgRNA3 2nd R	GTGACTGGAGTTCAGACGTGT GCTCTTCCGATCT CTAAGGAAATGATCATGCTGTC	
1b-sgRNA4 1st F	AATGCATATGTAGTGTCTATTGCA	
1b-sgRNA4 1st R	TAGCATTCAGCAACAAAGCT	
1b-sgRNA4 2nd F	ACACTCTTTCCCTACACGAC GCTCTTCCGATCT TTTGATCTTGTGCCATCTGATC	
1b-sgRNA4 2nd R	GTGACTGGAGTTCAGACGTGT GCTCTTCCGATCT CTGTTAGTGTCCTGCTGCTA	
1ab-sgRNA3 1st F1	GTTTGAGCTGCTATCGATTGTT	Targeted deep-sequencing
1ab-sgRNA3 1st F2	GTTTGAGCTGCTATCGATTGTT	
1ab-sgRNA3 1st R1	AAGCATTGAGCGCAAACTAAG	
1ab-sgRNA3 1st R2	AAGCATTGAGCGCAAACTAAG	
1ab-sgRNA3 2nd F1	ACACTCTTTCCCTACACGAC GCTCTTCCGATCT TTTGATCTTGTGGGATCTGATC	
1ab-sgRNA3 2nd F2	GTGACTGGAGTTCAGACGTGT GCTCTTCCGATCT TTTGATCTTGTGGGATCTGATC	
1ab-sgRNA3 2nd R1	ACACTCTTTCCCTACACGAC GCTCTTCCGATCT GAGTATTGTGGATGGCTGTTT	
1ab-sgRNA3 2nd R2	GTGACTGGAGTTCAGACGTGT GCTCTTCCGATCT GAGTATTGTGGATGGCTGTTT	
